# Association between Serum Ferritin Concentrations and Depressive Symptoms among Chinese Adults: A Population Study from the Tianjin Chronic Low-Grade Systemic Inflammation and Health (TCLSIHealth) Cohort Study

**DOI:** 10.1371/journal.pone.0162682

**Published:** 2016-09-09

**Authors:** Qian Su, Yeqing Gu, Bin Yu, Fei Yu, Haiyan He, Qing Zhang, Ge Meng, Hongmei Wu, Huanmin Du, Li Liu, Hongbin Shi, Yang Xia, Xiaoyan Guo, Xing Liu, Chunlei Li, Xue Bao, Fangfang Liu, Liyun Fang, Huijun Yang, Shaomei Sun, Xing Wang, Ming Zhou, Qiyu Jia, Honglin Zhao, Kun Song, Kaijun Niu

**Affiliations:** 1 Nutritional Epidemiology Institute and School of Public Health, Tianjin Medical University, Tianjin, China; 2 Health Management Centre, Tianjin Medical University General Hospital, Tianjin, China; 3 Institute of Psychology, Tianjin Medical University, Tianjin, China; 4 Collaborative Innovation Center of Non-communicable Disease, Tianjin Medical University, Tianjin, China; University of Illinois at Urbana-Champaign, UNITED STATES

## Abstract

Depressive symptoms have become the most important global public health issue. Iron plays an important role in brain function, cognition, and behavior, and its impacts on depressive symptoms may be multifactorial with both positive and negative effects. Previous observational studies focusing on the association between iron status and depressive symptoms showed inconsistent results. Ferritin is a ubiquitous intracellular protein that can store and release iron and is widely used as a clinical biomarker to evaluate iron status. We performed a cross-sectional study to examine the relationship between serum ferritin and depressive symptoms among 3,839 subjects who were from the Tianjin Chronic Low-grade Systemic Inflammation and Health (TCLSIHealth) cohort. Depressive symptoms were assessed using the Chinese version of 20-item self-rating Depression Scale (SDS) with 4 cutoffs (40, 45, 48 and 50) to indicate elevated depressive symptoms (40 was the primary cut-off). The prevalence of depressive symptoms was 36.5%, 17.6%, 11.0% and 7.0% for SDS ≥40, ≥45, ≥48 and ≥50, respectively. With the primary cut-off point of 40, multiple potential confounding factors were adjusted and the odds ratios (95% confidence interval) of having elevated depressive symptoms by quartiles of serum ferritin concentrations were 1.00 (reference), 1.10 (0.91, 1.34), 0.81 (0.66, 1.01) and 1.02 (0.81, 1.28) for the first, second, third and fourth quartile, respectively (*P* for trend = 0.76). Similar relations were observed with the use of other cut-offs as a definition of depressive symptoms. In conclusion, there is no significant relationship between serum ferritin concentrations and depressive symptoms among Chinese adults.

## Introduction

Depressive symptoms have a high prevalence [1] and are closely associated with physical health [2], social function, quality of life [3, 4], and attempted suicide [5, 6]. Moreover, depressive symptoms have been reported to be the leading cause of mortality and morbidity, meaning they carry a considerable disease burden [7]. Thus, depressive symptoms have become the most important public health concern.

There is a compelling relationship between nutrition and depressive symptoms [8]. Iron status plays an important role in brain function, cognition, and behavior, and its impacts on depression may be multifactorial with both positive and negative effects [9]. Ferritin is a ubiquitous intracellular protein that can store and release iron and is widely used as a clinical biomarker to evaluate iron status [10]. On the one hand, iron deficiency, commonly characterized by decreased serum ferritin in the general population, is the most prevalent nutritional problem [11] related to social and economic development in both developing and developed countries [12, 13]. The many consequences of iron deficiency are alterations in mental and cerebral mechanisms, emotions, and behavior, resulting in mood disorders [9, 14–16]. On the other hand, prior studies in patients with depression suggest that systemic, low-grade inflammation plays a role in the onset and course of the disease [17–22]. Important determinants of an inflammatory process are characterized by decreased serum iron and transferrin levels with normal or increased ferritin levels [23].

Given the above concepts, it is important to understand the role of iron status in depression. However, previous observational studies focusing on the relationship between serum ferritin levels and depression have had limitations regarding their sample populations [23–32]. They only included clinical patients or selected subgroups of the general population even though depressive symptoms are more common in the general population than other forms of depression. Moreover, the results of these studies were inconsistent. Several previous studies showed that depressive symptoms were associated with decreased levels of serum ferritin concentrations or not associated at all, while increased levels of serum ferritin concentrations were observed only in clinical patients with major depression or post-stroke depression.

Hence, the present study aimed to examine the association between serum ferritin status and depressive symptoms in a large general population.

## Subjects and Methods

### Study participants

Participants in this study were recruited during 2013–2014 from the Tianjin Chronic Low-grade Systemic Inflammation and Health (TCLSIHealth) cohort, a prospective dynamic cohort focusing on the relationship between chronic low-grade inflammation and the health status of a population living in the Tianjin [33], a city located in the northeast of the North China Plain with approximately 10.43 million inhabitants. Subjects in the present study were sampled by a random process, using a random number generator. Nearly all occupations are covered in this study, and we also included retired individuals living in residential communities. Therefore, the sample population used here is representative of the general adult population in Tianjin. All participants were asked to fill in a structured self-administered health status questionnaire during their annual health examinations at the Health Management Centre of Tianjin Medical University General Hospital, the largest and most comprehensive physical examination center in Tianjin. The fasting blood sample was routinely drawn 12 ml of whole blood from each subject for 2 ml of plasma and 10 ml of serum.

**Fig 1:**
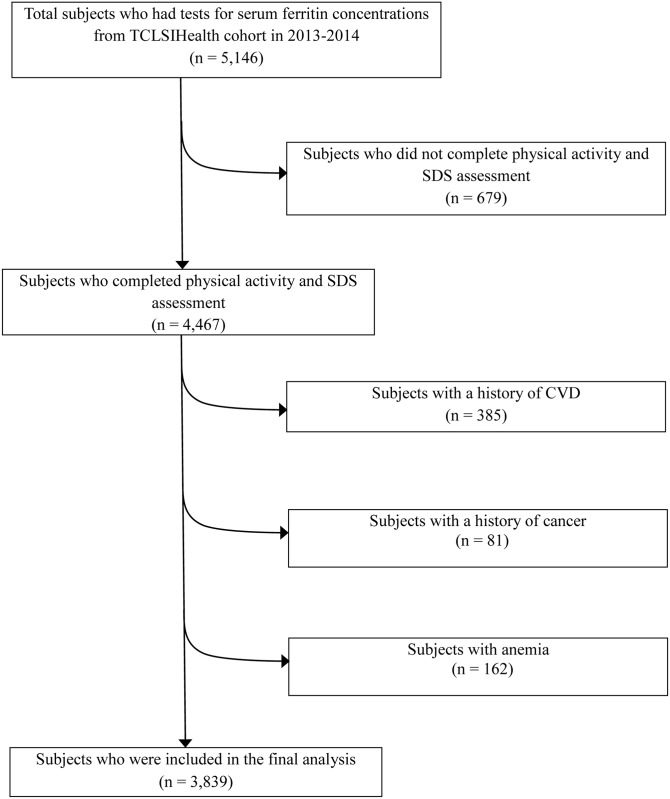
Flow chart of the sample selection. TCLSIHealth cohort: the Tianjin Chronic Low-grade Systemic Inflammation and Health cohort; SDS: the Zung Self-Rating Depression Scale; CVD: cardiovascular disease.

The baseline data during 2013–2014 was used to perform a cross-sectional analysis in this study. During the survey period there were 5,146 subjects who participated in the cohort and had tests for serum ferritin concentrations (Fig 1). We excluded participants with missing information on depression scale, body height and/or body weight measurements and physical activity (PA) (679), or those with a history of CVD (n = 385), cancer (n = 81) and anemia (n = 162). The final cross-sectional analyzed population comprised 3,839 participants (males, 59.5%) aged 48.5 years (SD (standard deviation), 11.6 years) after the exclusion.

The protocol of this study was approved by the Institutional Review Board of Tianjin Medical University and written informed consent was obtained from all participants. The study conforms to the strengthening the reporting of observational studies in epidemiology (STROBE) guidelines for cross-sectional study.

### Assessment of depressive symptoms

The assessment of depressive symptoms was performed using the Chinese version of the Zung Self-Rating Depression Scale (SDS), a useful questionnaire commonly recognized by Chinese psychiatrists with satisfactory reliability and validity [34, 35]. There were 20 items defined as either positive or negative, and the participants were required to score on a scale of 1 to 4 for each item. The total SDS score ranges from 20 to 80, with greater values indicating increased severity. In the present study, 4 cut-off points (40, 45, 48 and 50) were used to define depressive symptoms and 40 served as the primary cut-off. Scores higher than the cut-offs are indicators of elevated depressive symptoms [34, 36]. If a participant was taking antidepressants or had a history of depression, he or she was also considered to have depressive symptoms.

### Assessment of serum ferritin concentrations and anemia

Fasting blood samples were taken by venipuncture of the cubital vein in the morning after an overnight fast. The serum ferritin levels were carried out using the Quantitative Kit for Tumor Markers (Protein Chip-Chemiluminescence). According to the kit, the measurement range of the assay was: Ferritin, 5-600ng/ml. The intra- and inter-assay coefficients of variation (CV) were less than 15%.

Hemoglobin (Hb) concentration was measured using an electronic counter by the cyanmethemoglobin method, and the intra- and inter-assay coefficients of variation (CV) were less than 3%. Anemia is defined when hemoglobin concentration was <130g/L for men and <120 g/L for women [37].

### Assessment of other variables

The other information contained in the health status questionnaire included demographic and socioeconomic characteristics, physical health status, lifestyle and health-related habits. Marital status was classified as married or unmarried. The educational level was measured by asking the question “what is the highest degree you earned?” and was divided into 2 categories: College graduate or ≥College graduate. The subjects were also classified as living alone or living with others. Employment status was classified as either Managers or Professionals. Managers were consisted of civilian staff of government departments and public institutions and senior officials or cadres. And visiting friends was evaluated by the question of “do you visit your friends?”. Drinking status of the study population was defined as “everyday,” “sometime,” and “ex-drinker” and smoking status was defined as two categories: “smoker” or “ex-smoker” according to the responses to the survey in the questionnaires. Physical activity (PA) was evaluated by using the short form of the International Physical Activity Questionnaire (IPAQ) [38]. The questionnaire asked whether subjects had performed any type of activities during the previous week: walking; moderate activity (eg, household activity or child care); vigorous activity (eg, running, swimming, or other sports activities). Metabolic equivalent (MET) hours per week for a certain type were calculated by using corresponding MET coefficients (3.3, 4.0 or 8.0, respectively) according to the following formula: MET coefficient of activity × duration (hours/day) × frequency (days/week). Weekly MET-h served as the indicator of total physical activity levels, which was computed by combining separate hours for different activities. A detailed personal history of physical illness and current medications were noted from ‘‘yes” or ‘‘no” responses to relevant questions.

The anthropometric variables (eg, height and body weight) were measured by using a uniform protocol with participants wearing light clothing and no shoes. Body mass index (BMI) was computed as the weight in kilograms (kg) divided by square of the height (m^2^). Waist circumference was measured at the umbilical level with subjects standing and breathing normally. Blood pressure (BP) was measured twice at the upper left arm in a sitting position by using an automatic device (Andon, Tianjin, China) after 5 minutes of rest. The mean of these 2 measurements served as the BP value. Blood samples for the analysis of fasting blood sugar (FBS) and lipids level were collected in siliconized vacuum plastic tubes. The glucose oxidase method was used to measure FBS, triglycerides (TG) were measured using enzymatic methods, low-density lipoprotein cholesterol (LDL) was measured using the polyvinyl sulfuric acid precipitation method, and high-density lipoprotein cholesterol (HDL) was measured using the chemical precipitation method with reagents from Roche Diagnostics on an automatic biochemistry analyzer (Roche Cobas 8000 modular analyzer, Mannheim, Germany). Metabolic syndrome (MS) was defined based on the criteria of the American Heart Association scientific statements of 2009 [39]. Diabetes mellitus (DM) was defined as fasting blood glucose ≥ 126 mg/dl (7.0 mmol/l) or physician-diagnosed diabetes and/or current use of antidiabetic medications according to the 1999 World Health Organization criteria [40]. White blood cell (WBC) counts were measured using an automated hematology analyzer XE-2100 (Sysmex, Kobe, Japan) and expressed as ×1,000 cells/mm^3^.

### Statistical Analysis

All statistical analyses were performed by using the Statistical Analysis System 9.3 edition for Windows (SAS Institute Inc, Cary, NC). The serum ferritin concentrations was used as independent variable in quartiles and depressive symptoms were used as dependent variable. The data was described as the mean with 95% confidence interval (95%CI) or as percentages, and the differences in proportion and means of measured variables (covariates) according to the quartiles of serum ferritin concentrations were examined by analysis of variance for continuous variables and by logistic regression analysis for proportional variables. Multiple logistic regression analysis was used to evaluate the associations between the quartiles of serum ferritin concentrations and depressive symptoms initially, taking the lowest quartile category as a reference. For crude model, the analysis was conducted without any adjustment; then the analysis was adjusted for age, sex, and BMI; for multiple-adjusted model, the analysis was adjusted further, in addition to smoking and drinking habits, PA, educational level, employment status, household incomes, living alone, visiting friends, marital status, total energy intake, MS, diabetes, the history of inflammatory diseases, intake of eicosapentaenoic acid (EPA) + docosahexaenoic acid (DHA) and WBC counts. The final multivariate logistic analysis was performed with the forced entry of all factors considered to be potential covariates. A linear trend across the quartiles of serum ferritin concentrations was tested by using the median value of each quartile as an ordinal variable. In addition, interactions between the quartiles of serum ferritin concentration and sex, BMI, smoking status and menopausal status were tested by the addition of the cross product terms in the regression model. The tests for interactions between the quartiles of serum ferritin concentration and these potential confounders in the final models were not found to be significant (*P* value for interaction = 0.56, 0.36, 0.39 and 0.51). Further analysis was conducted in males and females, respectively. *P* values <0.05 was considered statistically significant and all tests presented were two-tailed.

## Results

### The characteristics of the subjects by the quartiles of serum ferritin concentration

The characteristics of the subjects by the quartiles of serum ferritin concentrations are shown in Table 1. Of 3,839 subjects, those with higher levels of serum ferritin concentrations were more likely to have MS and diabetes, smoke, drink alcohol, be older, male, and married, and have a higher BMI, income, and WBC counts (*P* for trend <0.0001). In addition, those subjects who had a lower education, lower levels of visiting friends or employ as Managers had higher levels of serum ferritin concentrations (*P* for trend <0.05). However, no significant differences were observed on living alone, intake of EPA + DHA, physical activity and the history of inflammatory diseases.

**Table 1:** Participant characteristics according to the quartiles of serum ferritin concentrations (n = 3,839) ^a^.

	The quartiles of serum ferritin concentrations (ng/ml, range)				*P* for trend ^b^
	Level 1	Level 2	Level 3	Level 4	
Characteristics	(1.0–46.2)	(46.3–74.9)	(75.0–130.4)	(130.5–1213.8)	-
No. of subjects	960	960	959	960	
Age (y)	44.6 (43.9, 45.3)	46.4 (45.7, 47.1) ^c^	48.5 (47.8, 49.3)	48.9 (48.1, 49.7)	< 0.0001
Sex (males, %)	23.4	47.7	75.6	91.3	< 0.0001
BMI (kg/m^2^)	23.7 (23.5, 23.9)	24.5 (24.3, 24.7)	25.7 (25.5, 25.9)	26.5 (26.3, 26.7)	< 0.0001
WBC counts (×1,000cells/mm^3^)	5.32 (5.23, 5.4)	5.5 (5.42, 5.59)	5.66 (5.57, 5.75)	5.92 (5.83, 6.01)	< 0.0001
Metabolic syndromes (yes, %)	18.2	28.3	37.0	52.7	< 0.0001
Diabetes (yes, %)	2.9	4.8	6.5	10.0	< 0.0001
Physical activity (Mets × hour/week)	9.5 (8.7, 10.4)	11.1 (10.1, 12.1)	10.2 (9.4, 11.2)	11 (10, 12)	0.24
Total energy intake (kcal/d)	2201.1 (2134.8, 2269.6)	2250.8 (2183, 2320.8)	2304.8 (2235.3, 2376.5)	2260.3 (2192.1, 2330.6)	0.04
Intake of EPA + DHA (g/d)	1.09 (1.09, 1.10)	1.09 (1.08, 1.09)	1.10 (1.09, 1.10)	1.11 (1.10, 1.11)	0.20
Smoking status (%)					
Smoker	10.8	18.7	31.8	43.3	< 0.0001
Ex-smoker	4.18	6.8	9.4	12.8	< 0.0001
Drinker (%)					
Everyday	2.6	4.6	8.3	15.3	< 0.0001
Sometime	50.0	58.1	63.4	64.0	< 0.0001
Ex-drinker	8.2	6.4	7.5	7.8	0.73
Marital status (married, %)	92.1	93.1	95.6	96.8	< 0.0001
Living alone (yes, %)	6.3	7.5	9.1	6.3	0.84
Education (≥ College graduate, %)	59.4	58.2	56.2	53.1	< 0.01
Working status (%)					
Managers	53.4	52.4	51.2	46.4	< 0.01
Professionals	12.5	16.7	16.2	13.3	0.66
Household income (≥ 10,000 Yuan, %)	37.7	34.3	40.0	51.6	< 0.0001
Visiting friends (yes, %)	67.6	66.2	66.4	62.3	0.02
History of inflammatory diseases (%)	3.4	4.8	4.6	2.5	0.07

^a^ BMI, body mass index; WBC, white blood cell; EPA, eicosapentaenoic acid; DHA, docosahexaenoic acid.

^b^ Analysis of variance or logistic regression analysis.

^c^ Least square geometric mean (95% confidence interval) (all such values).

### The relationship between serum ferritin concentrations and depressive symptoms

The relationship between the quartiles of serum ferritin concentrations and depressive symptoms with logistic regression is presented in Table 2. The prevalence of depressive symptoms was 36.5%, 17.6%, 11.0% and 7.0% for SDS 40, 45, 48and 50, respectively. The assessment of depressive symptoms was performed in 4 cut-offs (40, 45, 48, and 50) and no significant association was found in crude and Age-, Sex- and BMI-adjusted and multiple-adjusted models with any cut-off. With the primary cut-off point of 40, the crude ORs (95% CI) of depressive symptoms associated with the quartiles of serum ferritin concentrations were 1.00, 1.05 (0.88, 1.27), 0.78 (0.65, 0.94) and 0.99 (0.82, 1.19) (*P* for trend = 0.47). Age-, Sex- and BMI-adjusted ORs (95% CI) for depressive symptoms across the quartiles were 1.00, 1.09 (0.90, 1.31), 0.83 (0.68, 1.03) and 1.07 (0.86, 1.34) (*P* for trend = 0.75). A variety of potential confounders were considered in the final multivariate logistic model, and the adjusted ORs for depressive symptoms were 1.00, 1.10 (0.91, 1.34), 0.81 (0.66, 1.01) and 1.02 (0.81, 1.28) (*P* for trend = 0.76). The results for the use of other cut-off points as a definition of depressive symptoms were essentially the same as those for the 40 cut-off point. In the multiple-adjusted model, the ORs (95% CI) of depressive symptoms for SDS 45, 48 and 50 were 1.00, 1.03 (0.81, 1.32), 0.85 (0.65, 1.11) and 1.01 (0.76, 1.34) (*P* for trend = 0.94); 1.00, 1.01 (0.76, 1.36), 0.84 (0.61, 1.17 and 0.81 (0.57, 1.16) (*P* for trend = 0.17); 1.00, 0.93 (0.65, 1.34), 0.93 (0.62, 1.38) and 0.89 (0.57, 1.37) (*P* for trend = 0.65), respectively (data not shown).

**Table 2:** The relationships of the quartiles of serum ferritin concentrations to depressive symptoms (n = 3,839) ^a^.

	The quartiles of serum ferritin concentrations (ng/ml, range)				*P* for trend ^b^
	Level 1	Level 2	Level 3	Level 4	
Adjusted odds ratio (95% CI)	(1.0–45.3)	(45.4–72.4)	(72.4–127.2)	(127.4–1213.8)	-
No. of subjects	960	960	959	960	-
No. of depressive symptoms (SDS ≥40)	361	373	307	359	-
Crude	1.00	1.05 (0.88, 1.27) ^c^	0.78 (0.65, 0.94)	0.99 (0.82, 1.19)	0.47
Adjusted for age, sex and BMI	1.00	1.09 (0.90, 1.31)	0.83 (0.68, 1.03)	1.07 (0.86, 1.34)	0.75
Multiple-adjusted model ^d^	1.00	1.10 (0.91, 1.34)	0.81 (0.66, 1.01)	1.02 (0.81, 1.28)	0.76

^a^ SDS, self-rating depression scale.

^b^ Obtained by using multiple logistic regression analysis.

^c^ Adjusted odds ratio (95% confidence interval) (all such values).

^d^ Adjusted for age, sex, body mass index, smoking status, drinking status, physical activity, marital status, total energy intake, household incomes, employment status, educational levels, visiting friends, living alone, metabolic syndrome, diabetes, history of inflammatory diseases, intake of EPA + DHA and white blood cell counts.

Furthermore, the results still remained insignificant, when the analysis was conducted in males and females, respectively. With the cut-off of 40, the multiple-adjusted ORs for depressive symptoms were 1.00, 1.15 (0.82, 1.62), 0.82 (0.59, 1.14) and 11.04 (0.75, 1.43) (*P* for trend = 0.94) in males and 1.00, 1.07 (0.84, 1.37), 0.81 (0.57, 1.14) and 1.17 (0.70, 1.92) (*P* for trend = 0.88) in females, respectively (data not shown).

## Discussion

With the aim to examine the association between serum ferritin concentrations and depressive symptoms in a large general population, the present study finds no significant association in multiple analyses.

Most of studies focusing on the link between serum ferritin and depressive symptoms found no significant association [25, 26, 28, 29]. The present study also found that there was no significant association between serum ferritin concentrations and depressive symptoms with any cut-offs of SDS scale. An alternative explanation might be that the role of iron status on depression is due to the sum of its positive and negative effects. Decreased serum ferritin concentration associated with depressive symptoms indicated the possible role of iron in brain function and the onset of depressive symptoms [23, 24, 41]. Iron deficiency results in poor brain myelination and impaired monoamine metabolism, which produce not only deficits in memory/learning capacity and motor skills, but also emotional and psychological disorders [14, 15]. Meanwhile, increased serum ferritin concentration in clinical patients was associated with depressive symptoms [30–32], with the key explain of inflammation for the association. Normal or increased ferritin levels play roles in the onset and development of depressive symptoms, as important components of the inflammatory process [23]. Other hypotheses was that whether serum changes in iron status reflect iron uptake in the brain at all [25]. Experimental data suggest that brain iron uptake may be constitutive and independent of plasma transferrin, transferrin saturation, or regional brain iron concentration supporting a nontransferrin-bound iron uptake pathway [42].

Furthermore, the results still remained insignificant, when the analysis in this study was conducted in females and males, respectively. Nevertheless, a gender difference was observed in a Japanese population where there was a significant difference in the levels of serum ferritin concentrations between genders [28]. This study indicated a lower level of serum ferritin concentrations in females than in males and also showed a gender difference regarding the association of serum ferritin concentrations and depressive symptoms with a certain cut-off point.

It is worth noting that the present study has several strengths, such as large sample of general population and adjustment for considerable potential confounding factors that could influence the results. We adjusted for multiple potentially confounding factors in our analysis. First, studies have shown that serum ferritin is higher in males than in females [28, 43] and is associated with many other socio-economic and lifestyle characteristics [44], which in turn could contribute to the development of depressive symptoms. Second, serum ferritin is associated with the risk of obesity, diabetes, MS and other chronic diseases [10, 43], which in turn are well-recognized risk factors for depressive symptoms [45]. Finally, markers of inflammation and immune (such as WBC) play an important role in the depressive symptoms [46]. WBC as a significant inflammatory factor is both accessible and affordable [33, 47]. Thus, we added all the above factors into the final model. Even after adjusting for all potential confounders, it still showed no significant relationship between the serum ferritin concentrations and depressive symptoms in such a large general population.

However, there are still several limitations in the present study. First of all, the relationship between serum ferritin concentrations and depressive symptoms comes from a cross-sectional design which does not allow causal relationship. The serum ferritin concentrations is ascertained simultaneously with depressive symptoms and, therefore, results could be alternatively interpreted as a consequence of reverse causation bias, that is, depression may lead to decreased serum ferritin concentrations. There is a need to conduct more prospective studies to determine whether serum ferritin is associated with depressive symptoms. Second, even though considerable confounding factors have been taken into consideration, there are other residual factors, especially dietary factors [48], associated with iron status (serum ferritin) and depressive symptoms, interfering the relationship between them. The present study cannot exclude the possibility that such dietary components could be responsible for the observed association. However, the relationship with serum ferritin remained insignificant after controlling for intake of EPA + DHA. Moreover, more data about other biomarkers of iron status (eg. serum iron and total iron binding capacity) and iron intake should be considered when exploring the association between iron status and depressive symptoms, while this study couldn’t. More information and further studies are needed. Finally, the measurement of depressive symptoms depends on a validated scales rather than a clinical diagnosis; therefore, it is necessary to explore the association of serum ferritin and the depression in patients with clinical diagnosis.

In conclusion, the present study shows that there is no significant association between serum ferritin concentrations and depressive symptoms among Chinese adults.
